# Bax-inhibiting peptide attenuates bleomycin-induced lung injury in mice

**DOI:** 10.1242/bio.026005

**Published:** 2017-11-14

**Authors:** Kunihiro Suzuki, Toyoshi Yanagihara, Tetsuya Yokoyama, Takashige Maeyama, Saiko Ogata-Suetsugu, Masako Arimura-Omori, Hironori Mikumo, Naoki Hamada, Eiji Harada, Kazuyoshi Kuwano, Taishi Harada, Yoichi Nakanishi

**Affiliations:** 1 Research Institute for Diseases of the Chest, Graduate School of Medical Sciences, Kyushu University, Fukuoka, 812-8582, Japan; 2 Division of Respiratory Diseases, Department of Internal Medicine, Jikei University School of Medicine, Tokyo, 105-8461, Japan

**Keywords:** Bax, Lung injury, Apoptosis

## Abstract

Bax is a pro-apoptotic member of the Bcl-2 family of proteins, and plays a central role in mitochondria-dependent apoptosis. Several lines of evidence have implied that Bax is involved in both epithelial apoptosis and fibroblast proliferation in idiopathic pulmonary fibrosis; however, the mechanisms remain unknown. Bax-inhibiting peptide V5 (BIP-V5) exhibits membrane permeability and inhibits the activation of Bax.

The purpose of this study was to investigate whether the control of Bax activity by BIP-V5 reduces the degree of bleomycin-induced lung injury. C57BL/6J mice were administered bleomycin and BIP-V5 intratracheally on day 0. Bronchoalveolar lavage fluid and lung tissue were obtained on day 7. Human pulmonary alveolar epithelial cells (A549 cells) and mouse pulmonary alveolar epithelial cells (LA-4 cells) were stimulated with bleomycin to induce apoptosis.

Administration of BIP-V5 improved the survival rate and degree of bleomycin-induced lung injury by suppressing Bax activation in mice. BIP-V5 treatment decreased bleomycin-induced apoptosis of alveolar epithelial cell lines (A549 cells and LA-4 cells) by suppressing Bax activation. These results indicate that administration of BIP-V5 may constitute a novel therapeutic strategy against lung injury.

## Abstract (precis)

**Summary:** The inhibiting peptide for Bax, a pro-apoptotic member of the Bcl-2 family of proteins, ameliorates bleomycin-induced lung injury in mice via apoptosis suppression in epithelial cells.

## INTRODUCTION

Idiopathic pulmonary fibrosis (IPF) is a fatal disease defined as a specific form of chronic fibrosing interstitial pneumonia and has the histological appearance of usual interstitial pneumonia. The median survival of patients with IPF has been reported as 3–4 years from the onset of respiratory symptoms (American Thoracic Society and European Respiratory Society, 2002). Although the prognosis of this disease is relatively poor, the etiology of IPF remains unknown, and the efficacy of current therapeutic interventions is unsatisfactory (Behr, 2012; Richeldi et al., 2011). Therefore, a novel strategy for IPF treatment needs to be established.

Alveolar epithelial cells are known as the primary site of lung damage in pulmonary fibrosis (Kuwano et al., 2004). It is thought that recurrent and persistent epithelial damage and its insufficient repair disrupt normal epithelial–fibroblast interactions and evoke fibroblast proliferation and pulmonary fibrosis (Gross and Hunninghake, 2001). Apoptosis is observed in the lung epithelial cells of patients with IPF (Barbas-Filho et al., 2001; Kuwano et al., 1996) and may be involved in alveolar epithelium injury (Hagimoto et al., 1997; Maeyama et al., 2001). Therefore, alveolar epithelial cell apoptosis is a potential target in IPF.

The BCL-2 protein family plays a pivotal role in apoptosis, which is a cell suicide system that is essential for development, tissue homeostasis, and immunity (Czabotar et al., 2014). Bcl-2 family members act as anti- or pro-apoptotic regulators, producing hetero- or homodimers. Bcl-2-associated X protein (Bax) is the main pro-apoptotic member of the Bcl-2 family, and is involved in mitochondria-dependent apoptosis. In healthy cells, Bax is located in the cytosol and, after the initiation of apoptotic signaling, Bax changes its conformation, produces homodimers and translocates to the mitochondrial membrane. This translocation results in the release of cytochrome c, followed by the activation of the mitochondrial apoptosis pathway. Various factors, including heat and hydrogen peroxide, stimulate Bax activation. In addition, Bax can be activated by binding to other pro-apoptotic Bcl-2 family proteins, such as BH3-only proteins (Bid, Bim, Noxa, PUMA, etc.). The expression of Bax is upregulated by p53, and Bax has been reported to be involved in p53-mediated apoptosis (Plataki et al., 2005).

The expression of Bax is increased in the pulmonary alveolar epithelial cells of patients with IPF (Plataki et al., 2005). Attenuation of lung fibrosis has been shown in Bax knockout mice (Kang et al., 2007); therefore, Bax may constitute a potential therapeutic target of alveolar epithelial injury in IPF.

Bax-inhibiting peptide V5 (BIP-V5) is a cell-permeable pentapeptide, which was created to mimic Ku70, which is a subunit of Ku, a DNA-dependent protein kinase holoenzyme, which binds Bax through residues 578–583 (Yoshida et al., 2004). BIP-V5 has a Bax-binding domain and acts to prevent the activation of Bax in the cytosol. Similar to Ku70, BIP-V5 interacts with Bax and prevents its conformational change (Gomez et al., 2007). In the present study, we investigated the roles of Bax in lung injury *in vivo* and *in vitro* using BIP-V5.

## RESULTS

### BIP-V5 attenuates bleomycin-induced lung injury in mice

**Fig. 1.**
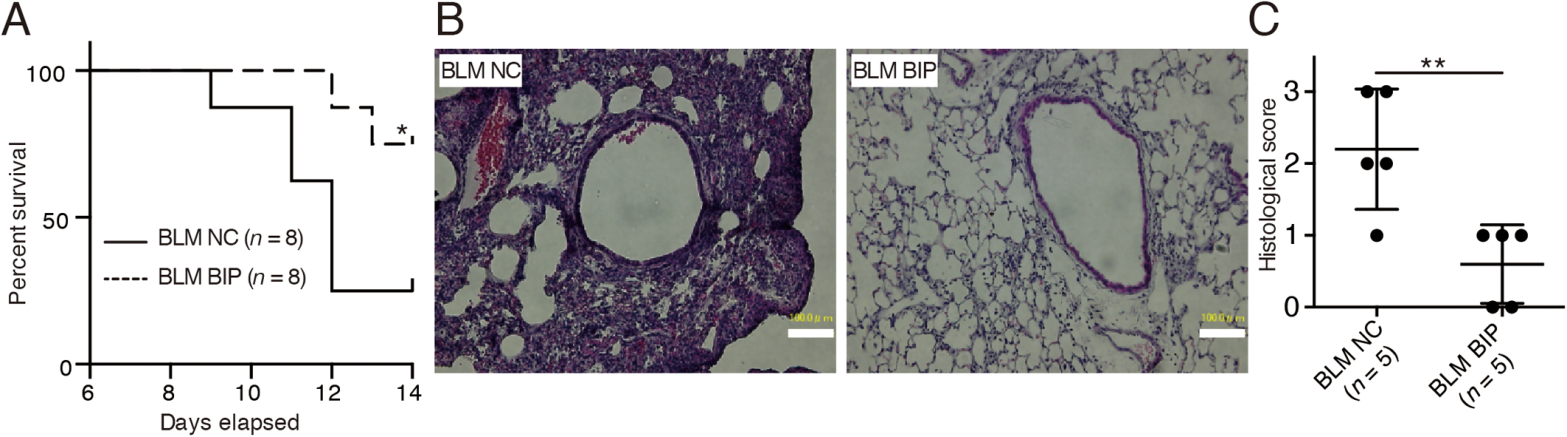
**BIP-V5 attenuates bleomycin-induced lung injury.** (A) Kaplan–Meier curve analysis with BLM+NC or BLM+BIP. (B) Hematoxylin and eosin-stained lung tissues from mice treated with BLM+NC or BLM+BIP on day 7. Scale bars: 100 µm. (C) The pathological grades of the tissues after hematoxylin and eosin staining. The pathological grades were determined according to the following criteria: 0, no lung abnormalities; 1, presence of inflammation and fibrosis involving <25% of the lung parenchyma; 2, lesions involving 25–50% of the lung; and 3, lesions involving >50% of the lung. *P<0.05, **P<0.01. NC, negative control; BLM, bleomycin; BIP, Bax-inhibiting peptide V5.

**Fig. 2.**
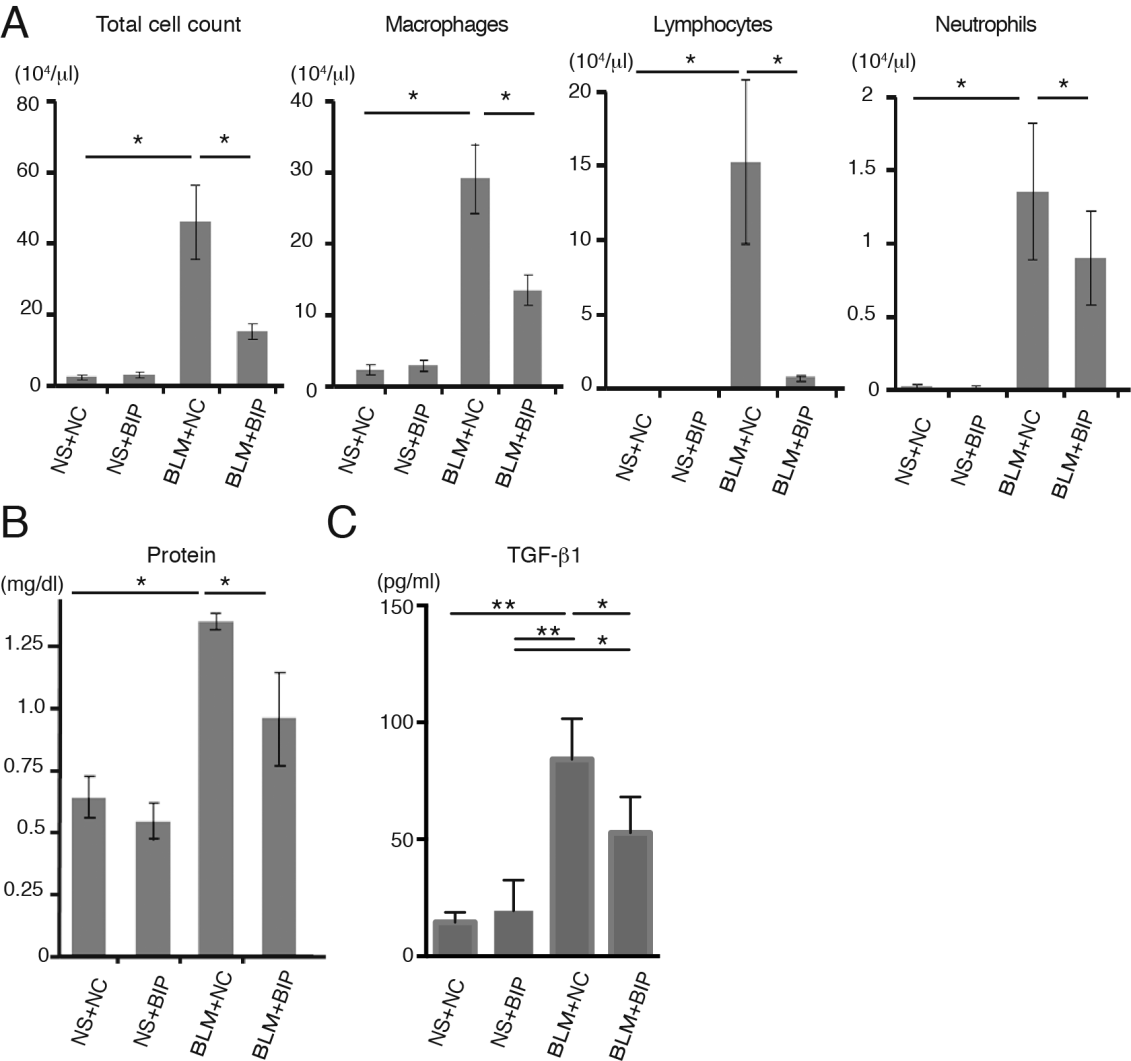
**BIP-V5 attenuates bleomycin-induced lung inflammation and TGF-β1 secretion.** (A) Total cell count and number of macrophages, lymphocytes and neutrophils in BALF on day 7. (B) Total protein concentration and TGF-β1 concentration in BALF on day 7. Data are presented as the mean±s.e.m. from three (A) or five (B,C) mice per group. *P<0.05, **P<0.01. NS, normal saline.

The survival of the mice treated with bleomycin (BLM) and BIP-V5 was significantly better than that of the mice in the BLM and negative control (NC) group (14-day cumulative survival rate, 75% versus 25%, respectively) (Fig. 1A). Extreme inflammatory cell infiltration in the lung interstitium, thickening of the alveolar septa, collapse of alveolar spaces, and proliferation of fibroblasts were observed on day 7 after BLM and NC instillation (Fig. 1B). BIP-V5 treatment alleviated all of these changes (Fig. 1B), and also decreased the pathological grade of inflammation (Fig. 1C). On day 7, the protein concentration and total number of cells in the bronchoalveolar lavage fluid (BALF) of mice treated with BLM and BIP-V5 were significantly lower than those in the BALF of the mice treated with BLM and NC (Fig. 2A,B). The transforming growth factor (TGF)-β1 levels in the BALF of mice treated with BLM were significantly increased. BIP-V5 treatment significantly decreased BLM-induced TGF-β1 levels in the BALF (Fig. 2C).

### BIP-V5 suppresses apoptosis in bleomycin-induced lung injury

**Fig. 3.**
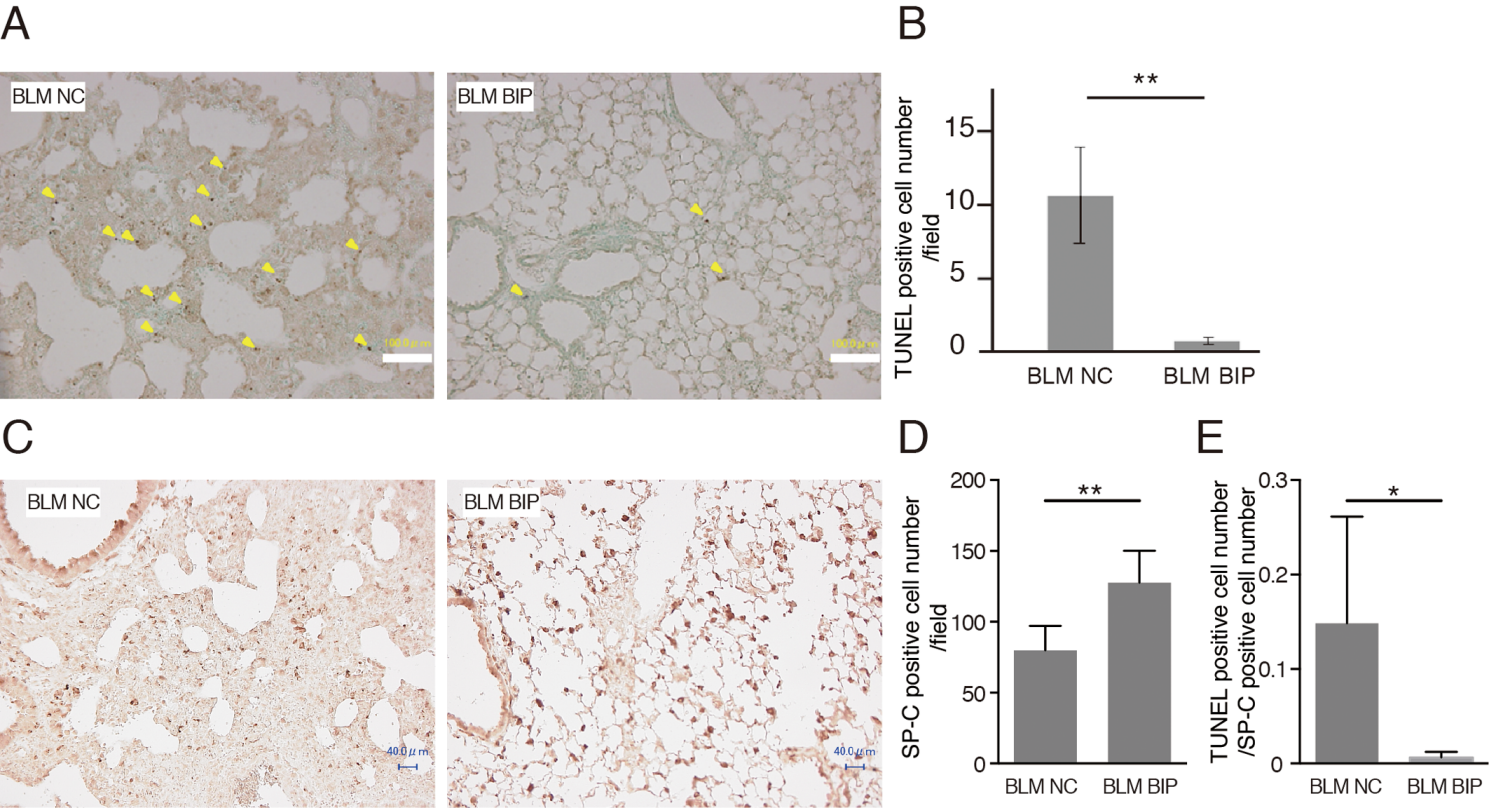
**BIP-V5 decreases apoptosis during bleomycin-induced pneumopathy.** (A) Representative images after TUNEL staining of the lung tissues from the BLM+NC and BLM+BIP groups on day 7. The arrowheads indicate evidence of apoptosis, including chromatin condensation. Scale bars: 100 µm. (B) Quantitative results of the number of TUNEL-positive cells in the lung tissues. (C,D) Immunohistochemical analysis of SP-C expression (C) and the quantitative results of SP-C-positive cell number (D) on day 7. TUNEL-positive cells and SP-C-positive cells were counted in all fields (40–55 fields) of the lungs using a light microscope with 200× magnification. Scale bars: 40 µm. (E) The ratio of TUNEL-positive cells to SP-C-positive cells. Data are presented as the mean ±s.e.m. from five mice per group. *P<0.05, **P<0.01.

The number of apoptotic cells detected using terminal deoxynucleotidyl transferase-mediated dUTP nick-end labeling (TUNEL) staining is considered to reflect the degree of lung injury (Kuwano et al., 1999). Some bronchiolar and alveolar epithelial cells and cells in inflammatory lesions revealed evidence of apoptosis as evaluated by TUNEL staining on day 7 after BLM instillation. The number of TUNEL-positive cells in BIP-V5-treated mice was significantly lower than that in NC-treated mice (Fig. 3A,B). The total number of cells was quite different between NC and BIP with BLM. However, the cells that increased were mainly inflammatory cells, and the TUNEL-positive cells were mainly epithelial cells. Thus, we counted the surfactant protein-C (SP-C)-positive cells, which indicate type II alveolar epithelial cells (Fig. 3C,D), and TUNEL-positive cell counts were normalized using SP-C-positive cell counts in the lung fields. Even after normalization using the SP-C-positive cell count, TUNEL-positive cell counts were lower when treated with BIP-V5 and BLM than when treated with NC and BLM (Fig. 3E).

### BIP-V5 suppresses epithelial cell apoptosis *in vitro*

**Fig. 4.**
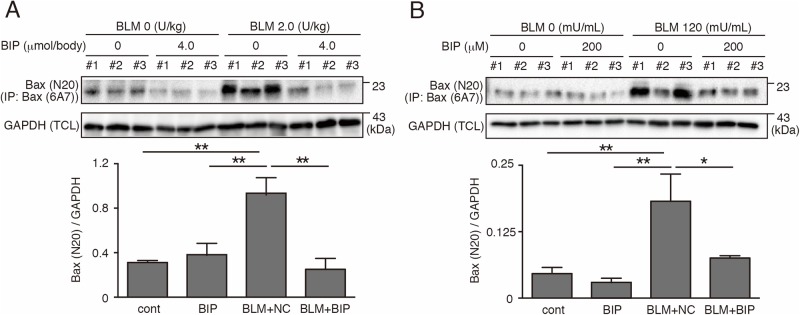
**BIP-V5 inhibits Bax activation in bleomycin-induced pneumopathy and bleomycin-treated A549 cells.** (A) The expression of the Bax-activated form of mouse bleomycin-induced pneumopathy homogenate lung on day 7. (B) The expression of Bax-activated form of A549 cells under BLM treatment. A549 cells were treated with normal saline or BLM (120 mU/ml) for 24 h. BIP-V5 (200 µM) was pre-dosed 1 h before the administration of saline or BLM. Data were collected from three separate experiments and are expressed as the mean±s.e.m. *P<0.05, **P<0.01. TCL, total cell lysate; IP, immunoprecipitation.

**Fig. 5.**
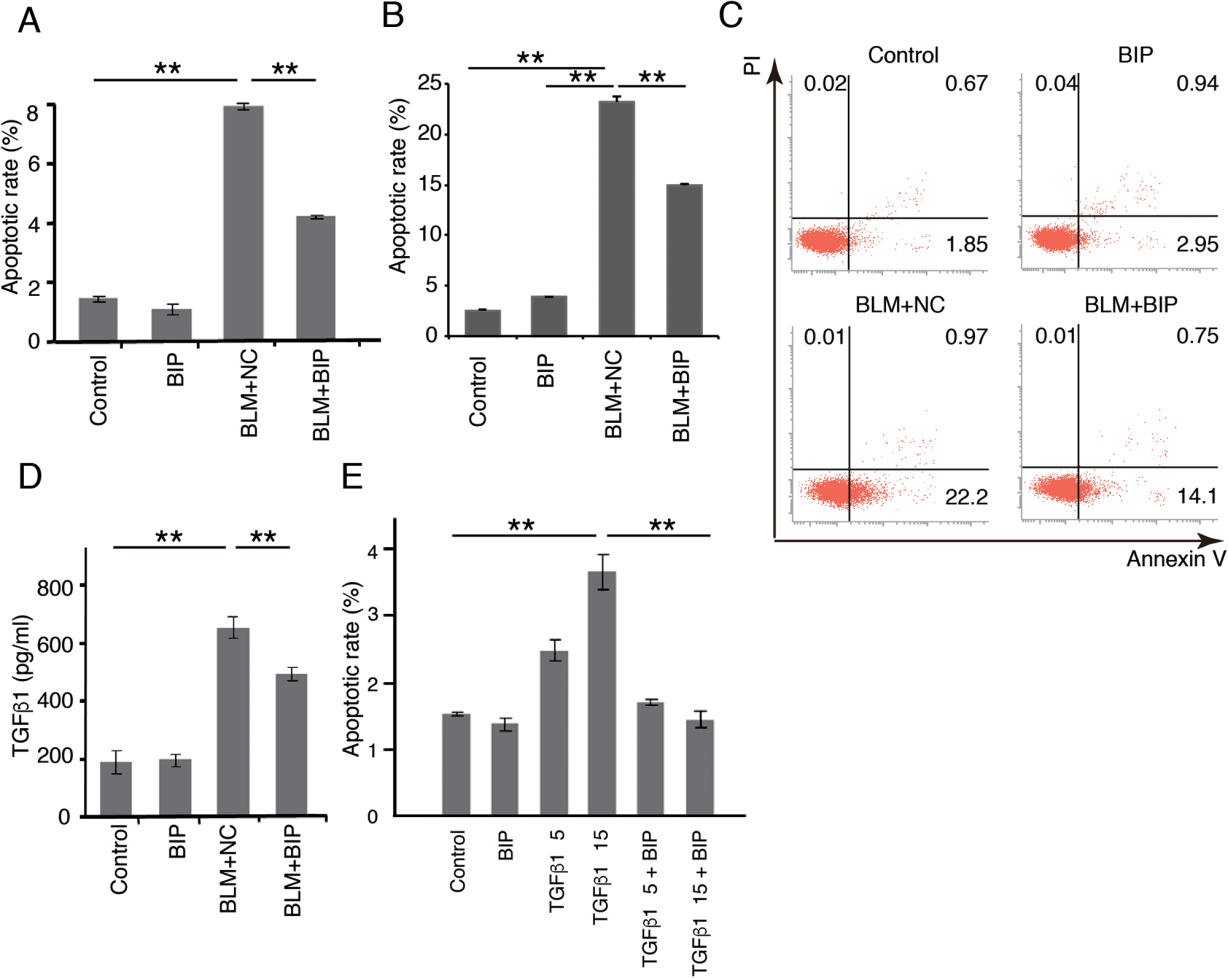
**Effect of BIP-V5 on the apoptosis of pulmonary alveolar epithelial cells.** (A) The apoptotic rate of A549 cells under BLM treatment. A549 cells were treated with normal saline or BLM (120 mU/ml) for 24 h. BIP-V5 (200 µM) was pre-dosed 1 h before the administration of saline or BLM. (B,C) The apoptotic rate of LA-4 cells under BLM treatment. LA-4 cells were treated with normal saline or BLM (120 mU/ml) for 12 h. BIP-V5 (200 μM) was pre-dosed 1 h before the administration of saline or BLM. Apoptotic rate (B) was measured by flow cytometry (C) as Annexin V positivity. (D) A549 cells were treated for 24 h with BLM. BIP-V5 (200 µM) was pre-dosed 1 h before the administration of BLM. TGF-β1 concentration in the supernatant of the medium was measured by ELISA. (E) The percentage of apoptotic cells under TGF-β1 stimulation. A549 cells were treated with normal saline or TGF-β1 (5 ng/ml or 15 ng/ml) for 72 h. BIP-V5 was pre-dosed 1 h before the administration of saline or TGF-β1. Data were collected from three separate experiments and are expressed as the mean±s.e.m. **P<0.01. PI, propidium iodide.

Activated Bax, which was detected by the anti-Bax 6A7 antibody, specifically recognizes conformational Bax protein changes (Yamaguchi and Wang, 2001). Activated Bax was significantly expressed in the lung tissues 7 days after BLM instillation, and was attenuated by BIP-V5 treatment (Fig. 4A). To further elucidate the relationship between Bax and apoptosis of alveolar epithelial cells, we used a human alveolar epithelial cell line (A549 cells). Activated Bax was significantly expressed in the cytoplasm of A549 cells 24 h after BLM instillation, and was attenuated by BIP-V5 treatment (Fig. 4B), same as *in vivo*. A549 cells underwent apoptotic changes 24 h after exposure to BLM, and BIP-V5 pretreatment significantly decreased these apoptotic changes (Fig. 5A). We also showed the inhibiting effect of BIP-V5 on BLM-induced apoptosis in a mouse lung epithelial cell line, LA-4 (Fig. 5B,C).

**Fig. 6.**
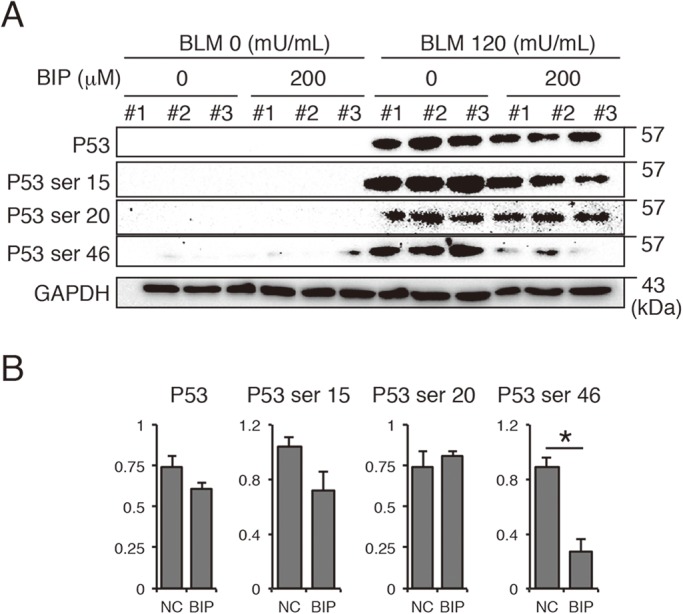
**Effect of BIP-V5 on p53 signaling.** (A) Western blot analysis of the phosphorylation of p53 in A549 cells stimulated with BLM. (B) Quantification of Western blotting. Each column represents the mean±s.e.m. (*n*=3). *P<0.05.

Furthermore, we assessed p53 phosphorylation at ser15, ser20 and ser46, which are required for the induction of apoptotic proteins. P53 is a protein present upstream of Bax and is also necessary in mitochondrial apoptosis (Plataki et al., 2005). Exposure to BLM upregulated the phosphorylation of p53 at ser15, ser20 and ser46, and BIP-V5 treatment significantly decreased the phosphorylation of p53 at ser46 (Fig. 6A,B).

Apoptosis-inducing conditions have been reported to increase the production of TGF-β1 (Solovyan and Keski-Oja, 2006). Therefore, alveolar epithelial cells were employed to verify whether the concentration of TGF-β1 was elevated under BLM stimulation. The concentration of TGF-β1 was elevated in the culture supernatants of A549 cells treated with BLM. The TGF-β1 concentration was significantly decreased following premedication with BIP-V5 (Fig. 5D). Furthermore, the A549 cells underwent apoptotic changes 24 h after stimulation with TGF-β1, and BIP-V5 pretreatment significantly decreased TGF-β1-induced apoptotic changes (Fig. 5E).

## DISCUSSION

A few studies have described the effect of local administration of BIP-V5 *in vivo* (Han et al., 2011; Wang et al., 2010). Because BIP-V5 is membrane permeable, dissolution in saline and subsequent intratracheal administration of BIP-V5 may have a direct effect on lung epithelial cells. We demonstrated that BIP-V5 has a protective effect on bleomycin-induced lung injury in mice. BIP-V5 suppressed Bax activity in bleomycin-treated mice lung tissues and bleomycin-treated alveolar epithelial cell lines. In addition, we confirmed that BIP-V5 suppressed expression of total Bax in bleomycin-treated mice lung tissues (Fig. S1); however, the reason was unclear.

*In vitro* experiments revealed that BIP-V5 suppressed BLM-induced apoptosis of A549 cells by inhibiting not only Bax activity but also p53 phosphorylation of ser46. P53 requires the phosphorylation of ser46, as well as ser15 and ser20, to induce pro-apoptotic proteins such as Bax. P53 not only acts as a transcriptional factor that regulates downstream target genes of Bax but also interacts directly with Bax (Selvakumaran et al., 1994; Westphal et al., 2014). This interaction between Bax and p53 may be related to BIP-V5 suppression of p53 phosphorylation of ser46 *in vitro*.

Various kinds of cytokines and growth factors participate in the pathological mechanism of IPF. TGF-β1 is one of the most important molecules involved in lung fibrosis (Higashiyama et al., 2007) and is produced from various kinds of cells, such as macrophages, lymphocytes, epithelial cells and mesenchymal cells (Khalil et al., 1993; Lo Re et al., 2011). In particular, epithelial cells produce TGF-β1 during apoptosis (Guo et al., 2012). TGF-β1 stimulation induces apoptosis in epithelial cells, and epithelial injury persists as long as TGF-β1 is produced. BIP-V5 inhibits TGF-β1 levels in BALF *in vivo*, TGF-β1 production, and TGF-β1-induced apoptosis in alveolar epithelial cells under BLM stimulation *in vitro*. Our results reveal that BIP-V5 has the potential to cease the vicious cycle of epithelial injury caused by TGF-β1.

In conclusion, the control of epithelial cell damage appears to be the key to therapeutic strategies against lung injury. Bax is suggested as an important intracellular molecule involved in both alveolar epithelial cell injury, and the administration of BIP-V5 may be a potent therapy against lung injury.

## MATERIALS AND METHODS

### Animal treatment

All experiments were conducted in accordance with the guidelines of the Animal Care and Use Committee of Kyushu University, and were approved by the Ethical Committee of Kyushu University Faculty of Medicine. Nine-week-old female C57BL/6J mice were purchased from Japan SLC, Inc. (Hamamatsu, Japan) and used in all experiments, except for a survival experiment in which 7-week-old females were used. After weighing, the mice were anesthetized with an intraperitoneal injection of ketamine sodium (Daiichi Sankyo Co. Ltd., Tokyo, Japan) and xylazine sodium (Wako Pure Chemical Industries Ltd., Tokyo, Japan), and were intratracheally administered 50 μl of bleomycin hydrochloride (Nippon Kayaku, Tokyo, Japan) in sterile saline. We used 2 U of bleomycin/kg body weight in all experiments. BIP-V5 (4 μmol/body weight; Merck Chemicals International, Kenilworth, NJ, USA) was intratracheally injected on day 0 with the bleomycin instillation. Control mice were injected with NC peptide (4 μmol/body weight; Merck Chemicals International) instead of BIP-V5. After the treatments, the mice were returned to their cages and allowed food and water *ad libitum*. The mice were anesthetized and sacrificed on day 7 after bleomycin instillation.

### Histopathology of lung tissues

Histopathology was performed as previously described (Hamada et al., 2008). The pathological grades were determined according to the following criteria: 0, no lung abnormalities; 1, presence of inflammation and fibrosis involving <25% of the lung parenchyma; 2, lesions involving 25–50% of the lung; and 3, lesions involving >50% of the lung.

### DNA damage and apoptosis in lung tissues

TUNEL staining was performed using a DeadEnd Colorimetric Apoptosis Detection System (Promega Corp., Madison, WI, USA) as previously described (Hamada et al., 2005).

### Immunohistochemistry of lung tissue

Immunohistochemistry was performed using a Histofine SAB-PO Kit (Nichirei, Tokyo, Japan). Anti-SP-C (FL-197) antibody (Santa Cruz Biotechnology, Dallas, TX, USA) was used as the first antibody. SP-C-positive cells were counted in all fields (40–55 fields) of the lung using a light microscope with 200-fold magnification.

### Bronchoalveolar lavage

The bronchoalveolar lavage method and analyses were performed as previously described (Hamada et al., 2008). Total protein concentrations in BALF were measured using the Bio-Rad Protein Assay (Bio-Rad Laboratories, Hercules, CA, USA).

### Measurement of TGF-β1 levels in BALF

BALF was centrifuged, and the supernatant was stored at −80°C until use. TGF-β1 levels in BALF were measured with a cytokine-specific enzyme-linked immunosorbent assay (ELISA) kit purchased from R&D Systems (Minneapolis, MN, USA).

### Cell culture and treatment

The A549 human lung epithelial cell line was cultured in Roswell Park Memorial Institute (RPMI) 1640 medium (Sigma Chemical Co., St. Louis, MO, USA) using 10% fetal bovine serum (FBS; Gibco BRL, Grand Island, NY, USA), penicillin and streptomycin in an incubator with 5% CO_2_ at 37°C. A549 cells were stimulated using bleomycin to induce apoptosis. The cells were pretreated with BIP-V5 before apoptotic stimulation. The cells were harvested 24 h after the addition of bleomycin and subsequently prepared for flow cytometry, immunoprecipitation and western blot.

The LA-4 murine lung epithelial cell line was cultured in F-12K (Kaighn's Modification of Ham's F-12 Medium) medium (Thermo Fisher Scientific, UK) using 10% FBS, penicillin, and streptomycin in an incubator with 5% CO2 at 37°C. LA-4 cells were stimulated using bleomycin to induce apoptosis (Stoner et al., 1975).

The cells were pretreated with BIP-V5 before apoptotic stimulation. The cells were harvested 12-24 h after the addition of bleomycin and subsequently prepared for flow cytometry.

### Flow cytometry

Apoptosis of A549 cells and LA-4 cells was assessed with Annexin V and propidium iodide (Roche Diagnostics, Basel, Switzerland). A549 cells were analyzed on an EPICS XL flow cytometer (Coulter, Luton, UK), and LA-4 cells were analyzed on a FACS Verse instrument (BD Bioscience).

### Western blot analysis

Cellular protein extract from mouse lung tissue and cell line were prepared by homogenization in radioimmunoprecipitation assay buffer. Proteins were separated using sodium dodecyl sulfate–polyacrylamide gel electrophoresis (SDS-PAGE). The proteins were transferred to hydrophobic polyvinylidene fluoride membranes (Millipore, Billerica, MA, USA), and the membranes were incubated using anti-Bax (N-20) antibody (Santa Cruz Biotechnology), anti-p53 (FL393) antibody (Santa Cruz Biotechnology), anti-GAPDH antibody (Cell Signaling Technology), anti-p53 ser15 antibody (Cell Signaling Technology), anti-p53 ser20 antibody (Cell Signaling Technology) and anti-p53 ser46 antibody (Cell Signaling Technology) in blocking buffer. After rinsing, the membranes were incubated with biotinylated anti-rabbit or mouse IgG. The blots were developed using the enhanced chemiluminescence method (Bio-Rad Laboratories). The signals were measured using ImageJ public domain software (https://imagej.nih.gov/ij/) and standardized to GAPDH.

### Immunoprecipitation followed by SDS-PAGE and immunoblotting

Homogenized A549 cells and mice lung tissues were incubated with Bax 6A7 antibody (Santa Cruz Biotechnology) for 6 h and mixed using rotary mixer NRC-200 (NISSEN, Tokyo, Japan) at 4°C temperature. Next, samples were incubated with protein G Mag Sepharose (GE Healthcare UK Ltd, Little Chalfont, Buckinghamshire, UK) for 6 h again. The antibody/antigen complex was pulled out of the sample using magnetic interaction between protein G Mag and Magrock6 (GE Healthcare UK Ltd). This isolated protein was separated by SDS-PAGE for western blot analysis. The Bax protein was detected using Bax N-20 antibody (Santa Cruz Biotechnology) after electrophoresis and transfer.

### Statistical analyses

Survival curves (Kaplan–Meier plots) were compared using the log-rank test. Pathological grades were compared using the Mann–Whitney *U* test. Comparisons of the numbers or contents of other results were carried out using the one-way ANOVA test followed by Tukey's multiple comparison test. *P* values less than 0.05 were considered to indicate statistical significance. All statistical analyses were performed using JMP Pro 10.0 (SAS Institute Japan, Tokyo, Japan).

## Supplementary Material

Supplementary information
