# Supplementary material for: Bax-inhibiting peptide attenuates bleomycin-induced lung injury in mice
Source: Biol Open. 2017 Nov 14;6(12):1869–75. doi: 10.1242/bio.026005 (PMC5769644; doi:10.1242/bio.026005)
Supplement: Supplementary information [file biolopen-6-026005-s1.pdf]

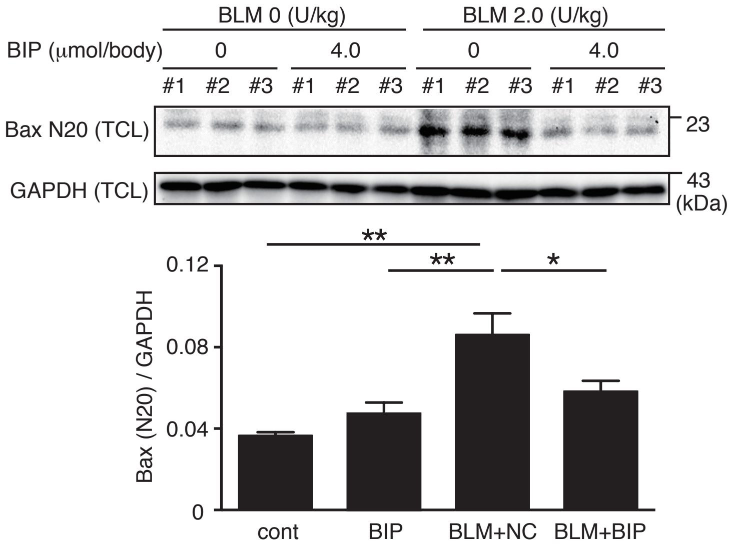

Fig. S1 Effect of Bax-inhibiting peptide V5 on the amount of total Bax expression in vivo.

(A) Western blot for total amount of Bax in lung tissues was performed on day 7. Data are collected from three separate experiments and are expressed as mean ± S.E.M. *$p < 0.05$, **$p < 0.01$. BLM, bleomycin; NC, negative control; BIP, Bax-inhibiting peptide V5.
